# Diffuse Purulent Bronchiectasy in Colts, with Morbid Anatomy Notes

**Published:** 1882-10

**Authors:** Wm. Osler, A. W. Clement

**Affiliations:** PROFESSOR AT MCGILL COLLEGE; LAWRENCE, MASS., STUDENT AT THE MONTREAL VETERINARY COLLEGE


### CASE DEPARTMENT.

### I.—DIFFUSE PURULENT BRONCHIECTASY IN A CALF, WITH MORBID ANATOMY NOTES.

REPORTED BY WM. OSLER, M.D., PROFESSOR AT MC GILL COLLEGE, AND A. W. CLEMENT, OF LAWRENCE, MASS., STUDENT AT THE MONTREAL VETERINARY COLLEGE.

The condition here described is stated to be infrequent in cattle, and affects old rather than young animals. It was doubtless induced by a bronchitis with copious secretions, which, lodging in the dependent tubes, caused dilatation and eventually infiltration of the walls, and extension of the inflammation to adjacent air cells. Professor Siedamgrolzky gives a plate (*Archiv. fur Thierheilkunde*, Bd. iv., Taf. iv.) exactly resembling the appearance in this instance.

A Galloway calf (female), aged six months, was admitted to the Infirmary of the Veterinary College on the morning of January 14th, 1882.

The following history was obtained: Healthy when born; it began to cough a few weeks after birth, and has ever since presented signs of chest trouble. It fed indifferently, and did not thrive. The family history is good. On examination, the animal was found to be much emaciated and very weak; coat rough and covered with lice; eyes sunken. It had a husky, frequent cough, without expectoration. Mucous membranes, congested. No response to pressure on trachea or larynx; pulse, 75 and weak; temperature, 102.6°; respiration, 38.

On percussion, a clear note was obtained except over a portion of left lung just behind the scapula, where there was perceptible dullness. On auscultation, the respiratory murmur could scarcely be detected over the portion corresponding to the area in which there was dullness. The animal was placed in a loose box and remained there until death, ten days after. The course of the disease during this period was a steady decline. The extremities and muzzle became cold; the abdomen, at times, tympanitic; the cough more violent, with slight but not fœtid expectoration.

The area of consolidation in the left lung increased, and there was slight dullness over anterior part of right lung. The respiration became hurried and weak, and on auscultation a harsh, rasping sound was heard, most evident over left lung. The temperature ranged from 100.6° to 104°, was irregular, usually an evening exacerbation.

*Autopsy.*—Much emaciation. The pathological condition was confined to the lungs. The anterior lobe in left lung was airless and dark in color, except in the upper part. The posterior lobe was crepitant and of natural color. There were no pleuritic thickenings or adhesions. On the affected portion of lung could be seen, beneath the pleura, numerous opaque white spots about the size of peas, not projecting beyond the membrane, often arranged in a foliaceous manner, and presenting irregular sinuous margins. These proved on subsequent examination to be greatly dilated bronchi, with inspissated contents. The appeaiance was best seen in the dependent portion of the lung. On section of the affected portion it appeared riddled with small and pretty closely set cavities—dilated bronchi—containing a tolerably consistent purulent matter of an opaque white color. These bronchi, when slit up with the probe-pointed scissors, proved to be in a state of dilatation, even to their ultimate ramifications. The intervening portion of lung-substance was of a dark livid red color and collapsed. There were no solid nodules, no extensive caseous masses, no cavities. The dilated and filled bronchi constituted the only important change in the organ. On tracing the larger bronchi of this lobe there also were found to contain muco-pus in considerable quantity. The upper part of this lobe was crepitant, and had no special alteration.

In the right lung a small area about a hands breadth in the anterior lobes was in the same condition, dark, livid in color, with the bronchi filled with a semi-caseous pus. The other three lobes were crepitant, and of good color.

The bronchial glands were soft and swollen, but not cheesy.

*Histology.*—The material contained in bronchial tubes consisted almost exclusively of pus cells in varying degrees of fatty degeneration. Mixed with these were a few larger cells. A portion of lung, frozen, sliced and stained in carmine showed the following: Under a low power were seen numerous irregular spaces which corresponded to the dilated bronchi, partially filled with purulent matter. In many places these have compressed the intervening parenchyma. With No. 7 (Hartnack) the walls of the distended bronchi were seen to have in great part lost their histological characters. The cilliated epithelium was nowhere visible, and the tissue appeared infiltrated with round cellular elements, often so crowded as to obscure even the fibrous tissue of the wall. In some the thickening of the wall was very marked, and there was the appearance of a central dilated lumen with thick infiltrated margins resembling the condition of nodular peribronchitis.

In several spots giant cells were found in the wall, but they were by no means abundant.

The whole condition as regards the bronchi appeared to be distention, with purulent contents, infiltration by a small celled growth of the bronchial walls and extension of this to a variable distance.

The tissue intervening between the distended bronchi was in great part collapsed. The air cells still visible, and did not present any marked epithelial proliferation. Here and there small aggregations of cells were deeply stained and there were giant cells in several of these.

